# Laser Acupuncture for Patients with Knee Osteoarthritis: A Systematic Review and Meta-Analysis of Randomized Placebo-Controlled Trials

**DOI:** 10.1155/2019/6703828

**Published:** 2019-11-03

**Authors:** Zhonggai Chen, Chiyuan Ma, Langhai Xu, Zhipeng Wu, Yuzhe He, Kai Xu, Safwat Adel Abdo Moqbel, Lidong Wu

**Affiliations:** Department of Orthopedic Surgery, The Second Affiliated Hospital, Zhejiang University School of Medicine, Hangzhou, Zhejiang 310000, China

## Abstract

**Objectives:**

To provide updated evidence from randomized controlled trials (RCTs) on the effectiveness of laser acupuncture for patients with knee osteoarthritis (KOA).

**Methods:**

A literature search in 9 databases was conducted from their inception through February 2019. Randomized controlled trials (RCTs) written in English that compared active laser acupuncture with placebo in KOA patients were included. Two authors independently extracted data from these trials. Meta-analysis software was used to analyze the data. Included studies were assessed in terms of the follow-up period, the methodological quality, and appropriateness of their technical features.

**Results:**

Of 357 studies, seven RCTs (totaling 395 patients) met the inclusion criteria. The short-term outcomes showed that laser acupuncture offered significant pain relief over placebo when assessed by the 100 mm visual analog scale (VAS) pain score (*p* = 0.02), while there was no significant difference between laser acupuncture and placebo based on Western Ontario and McMaster Universities Arthritis Index (WOMAC) pain score (*p* = 0.25). For subgroup analysis, laser acupuncture had superiority over placebo in terms of both VAS and WOMAC pain scores in the appropriate technical features subgroup and the excellent methodological quality subgroup. But the effect of laser acupuncture on pain relief was not maintained in terms of either VAS (*p* = 0.19) or WOMAC pain score (*p* = 0.60). The pooled effect showed no significant difference between laser acupuncture and placebo at either time point according to WOMAC function scale, WOMAC stiffness scale, and quality of life outcome.

**Conclusions:**

Our findings indicate that laser acupuncture can effectively reduce knee pain for patients with KOA at short term when appropriate technical features are applied, but the effect likely fades away during the subsequent follow-up period.

## 1. Introduction

Osteoarthritis (OA) is one of the most common degenerative diseases that cause chronic pain and disability in the elderly [1], with knee OA ranked 11^th^ among 291 diseases for disability globally [2]. Knee OA cannot be cured so far and will likely worsen over time [3], and increases in life expectancy and ageing populations are expected to exacerbate the healthcare burden. The management for knee OA mainly aims to relieve joint pain, improve joint function, and enhance the quality of life [4, 5]. Currently available modalities of management for knee OA include nonpharmacological, pharmacological, and surgical treatments [6]. During the past decade, much emphasis has been put on nonpharmacological management. It is widely recommended that the nonpharmacological intervention should be the first line of treatment for people with knee OA [6–8]. There are many modalities of this intervention, such as exercise, education, weight loss, manual therapy, acupuncture, bracing and taping, orthoses, balneotherapies, electrotherapies, and other complementary therapies [1, 8].

Laser acupuncture, defined as the stimulation of traditional acupuncture points with low-intensity laser irradiation [9], is one of the modalities of nonpharmacological intervention. Laser acupuncture is suggested to be a safer technique due to its noninvasive nature (e.g., in cases of HIV infection) and be a method which is more appropriate for the stimulation of difficult points such as auricular acupuncture points when compared to the traditional needling method [10, 11]. Therefore, laser acupuncture has been widely used to relieve pain in different musculoskeletal diseases. Although the therapeutic use of laser acupuncture is rapidly gaining in popularity, objective evaluation of its efficacy on knee OA in published studies is difficult because the treatment dosages and parameters applied in different studies vary quite a lot [9, 11]. Studies that use inappropriate dosages or inadequate parameters tend to get negative results [5, 11], causing that reported clinical therapeutic outcomes is conflicting.

Recently, there has been an increased number of randomized controlled trials (RCTs) evaluating the therapeutic efficacy of laser acupuncture on knee OA, and more trials conform to the guideline recommendations or expert consensus [12, 13]. Therefore, a systematic review with meta-analysis was conducted to update the previous systematic review and meta-analysis regarding laser acupuncture [10, 11], with the aim to evaluate the effectiveness of laser acupuncture on symptoms and function in patients with knee OA.

## 2. Methods

### 2.1. Search Strategy

The following nine databases were searched from their inception dates to February 2, 2019: MEDLINE, EMBASE, CINAHL, PubMed, Web of Science, Cochrane Library, Scopus, PEDro, and CNKI. In addition, the key journals (Lasers in Surgery and Medicine; Photo-medicine and Laser Surgery) were searched manually to cover recent studies, which may have not been included in other databases. Text words and controlled vocabulary (e.g., Medical Subject Heading) were used as keywords in the search. On the basis of the MEDLINE (Ovid) search strategy (Supplementary [Supplementary-material supplementary-material-1]), queries were revised to perform the best searches in the other databases.

### 2.2. Inclusion Criteria

Studies were considered eligible if they met the PICOS (population, intervention, comparator, outcome, and study design) criteria. Population: patients with knee OA (as assessed with radiography or according to the clinical criteria of the American College of Rheumatology guidelines), but knee arthroplasty has not been performed. Intervention: the experimental group received laser acupuncture treatments while the control group received sham laser acupuncture. Outcome: pain and/or functional outcomes of patients. Study design: RCTs.

### 2.3. Literature Selection

All relevant studies were imported into Endnote X8, and then duplicate studies were excluded. Next, the titles and abstracts of included studies were read by two reviewers independently, and the full-text articles were assessed for eligibility. At last, the studies that did not satisfy the PICOS were excluded. Disagreement about which studies to include was discussed by both reviewers until consensus was reached.

### 2.4. Assessment of Methodological Quality

Articles included in the review were assessed for methodological quality using the PEDro scale [14]. The PEDro scale has been accepted as a valid and reliable measure of the methodological quality of RCTs [15–17], and the PEDro score of each selected study can provide an indicator of the methodological quality (9–10 = excellent; 6–8 = good; 4–5 = fair; and <4 = poor). All included studies were assessed by two independent reviewers, and the discrepancy was resolved by consensus after discussion.

### 2.5. Data Extraction

Study data were extracted by two independent reviewers including study population, details of interventions, and outcome measures at different follow-up time points. If there were disagreements between two reviewers, a third reviewer was available to check for accuracy.

### 2.6. Outcome Measures

The primary outcomes of interest were the Western Ontario and McMaster Universities Arthritis Index (WOMAC) pain scores and the 100 mm visual analog scale (VAS) pain scores. The secondary outcomes of interest were the WOMAC stiffness and function scores and the quality of life questionnaire scores. If available, means and standard deviations for outcome measures were extracted or calculated according to the existing relevant data. Data extracted from outcomes were pooled for further meta-analysis.

### 2.7. Statistical Analysis

Meta-analysis was performed using Review Manager (RevMan) software, version 5.3 (Copenhagen: The Nordic Cochrane Centre, The Cochrane Collaboration, 2014). Means and standard deviations were used to calculate a standard mean difference (SMD) and 95% confidence interval (CI) in the meta-analysis because all the outcomes were continuous data. A negative SMD was defined to favor laser acupuncture to the control intervention and vice versa. Clinically relevant heterogeneity and statistical heterogeneity were assessed by the chi-square test (*p* < 0.05) and *I*^2^ statistics (≥50%), respectively [18]. A random-effects model was used for the main analyses.

### 2.8. Subgroup Analysis

Considering the changes in outcome data (difference between various time points in a study), outcome data were subgrouped according to the follow-up period. The best outcome data among measures taken within 2 months after the end of the intervention were regarded as the “short-term effect” of laser acupuncture in a special study, while the last outcome data among measures taken from 2 months to a year postintervention were regarded as the “long-term effect.” Besides, we analyzed the effect of laser acupuncture in subgroups distinguished by methodological quality and appropriateness of their technical features.

## 3. Results

### 3.1. Study Selection and Characteristics

**Figure 1:**
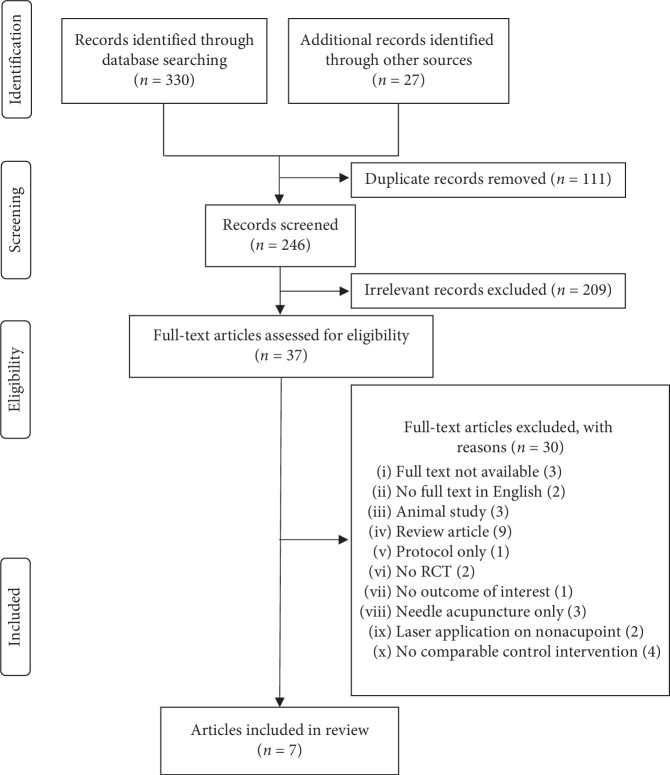
Flowchart showing the selection process of the review, based on the Preferred Reporting Items for Systematic Reviews and Meta-Analyses (PRISMA) statement.

Figure 1 illustrates the process of the study selection in this meta-analysis. In general, 330 potentially relevant articles were identified through database searching, and 27 extra studies were retrieved from the two key journals mentioned above. After removing the duplicate records, 246 articles remained. Then, 209 of these articles were excluded based on the title and abstract content. The remaining 37 articles were assessed for eligibility, and another 30 articles were excluded for reasons. Lastly, a total of 7 studies were included in this review.

Table 1 describes the characteristics of all 7 included RCT studies, which enrolled a total of 395 KOA patients: 203 patients in the active laser acupuncture group and 192 patients in the placebo laser acupuncture group. The placebo design was implemented by an inactive probe inside the laser arm in each included study, which was undetectable to the patients; this provided a credible sham comparator for laser acupuncture. However, the technical features of laser acupuncture treatments were diverse in terms of laser parameters and acupuncture points, as shown in Table 2. All available outcome measures of interest were extracted and analyzed in the meta-analysis.

### 3.2. Methodological Quality

The methodological quality of the studies was generally high (Supplementary [Supplementary-material supplementary-material-1]). According to the PEDro scale [14], the most recent two studies [19, 20] were considered to be of “excellent quality” while the other five studies [21–25] were classified as “good quality.” As shown in Supplementary [Supplementary-material supplementary-material-1], patients and assessors were blinded successfully in all the studies; however, only four out of the 7 studies provided adequate follow-up data with <15% dropout rate.

### 3.3. Meta-Analysis

#### 3.3.1. Primary Outcomes: Effects of Laser Acupuncture on Pain Relief

**Figure 2:**
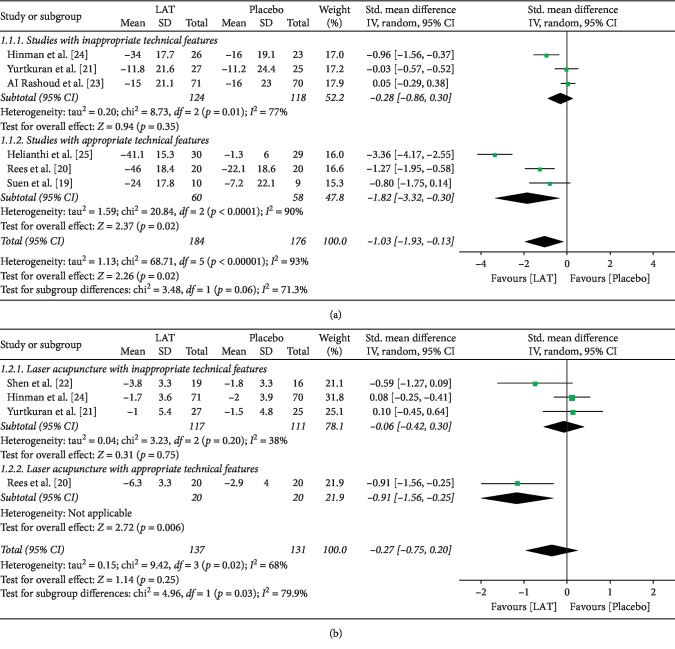
Forest plots of the LAT effects on short-term pain relief. (a) Pain relief regarding the VAS pain score (subgroup analysis based on whether studies with appropriate technical features). (b) Pain relief regarding the WOMAC pain score (subgroup analysis based on whether studies with appropriate technical features). LAT, laser acupuncture treatment; CI, confidence interval; SD, standard deviation.

At the best time point within 2 months after the end of the treatment, laser acupuncture was superior to placebo when assessed by the 100 mm VAS pain score (SMD = −1.03 (95% CI = −1.93, −0.13), *I*^2^ = 93%, *p*=0.02) (Figure 2(a)), but it was not superior when assessed by the WOMAC pain score (SMD = −0.27 (95% CI = −0.75, 0.20), *I*^2^ = 68%, *p*=0.25) (Figure 2(b)). Subgroup analysis based on whether the technical features of laser acupuncture treatments were appropriate showed that laser acupuncture had superiority over placebo in terms of both VAS and WOMAC pain scores in the appropriate technical features subgroup (SMD = −1.82 (95% CI = -3.32, −0.31), *I*^2^ = 90%, *p*=0.02; SMD = −0.91 (95% CI = −1.56, −0.25), *p*=0.006) (Figure 2). Moreover, another subgroup analysis based on methodological quality got the similar results in the excellent quality subgroup (SMD = −1.11 (95% CI = −1.66, −0.55), *I*^2^ = 0%, *p* < 0.0001; SMD = −0.91 (95% CI = −1.56, −0.25), *p*=0.006) (Supplementary [Supplementary-material supplementary-material-1]).

**Figure 3:**
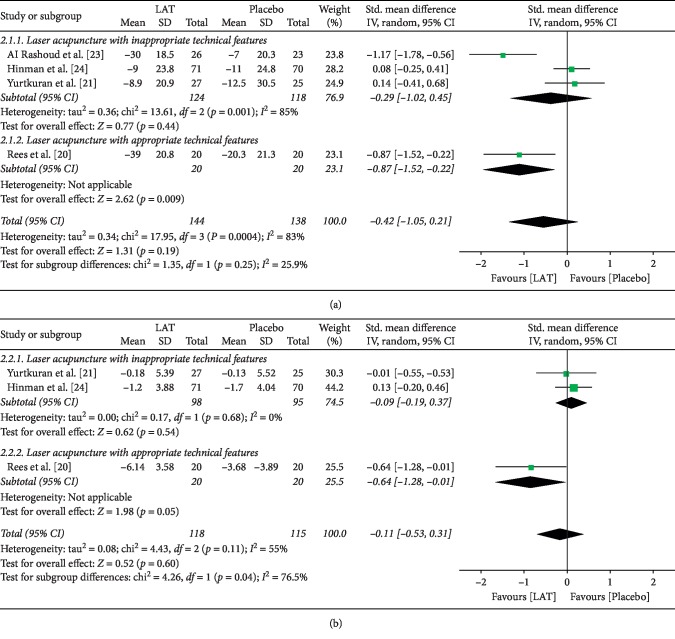
Forest plots of the LAT effects on long-term pain relief. (a) Pain relief regarding the VAS pain score (subgroup analysis based on whether studies with appropriate technical features). (b) Pain relief regarding the WOMAC pain score (subgroup analysis based on whether studies with appropriate technical features). LAT, laser acupuncture treatment; CI, confidence interval; SD, standard deviation.

When analyzing the long-term effect of laser acupuncture treatment, the overall effect on pain relief did not favor laser acupuncture in terms of either VAS or WOMAC pain score (SMD = −0.42 (95% CI = −1.05, 0.21), *I*^2^ = 83%, *p*=0.19 (Figure 3(a)); SMD = −0.11 (95% CI = −0.53, 0.31), *I*^2^ = 83%, *p*=0.60 (Figure 3(b))). Subgroup analysis was performed according to the appropriateness of technical features used in studies, and only one study [20] conformed to the guideline recommendations or expert consensus, which showed favorable effect of laser acupuncture on long-term pain relief in terms of both VAS and WOMAC pain scores (SMD = −0.87 (95% CI = −1.52, −0.22), *p*=0.009; SMD = −0.64 (95% CI = −1.28, −0.01), *p*=0.05) (Figure 3).

#### 3.3.2. Secondary Outcomes: Effects of Laser Acupuncture on WOMAC Function Score, Stiffness Score, and Quality of Life

Table 3 summarizes the results of meta-analysis for secondary outcomes. In detail, four studies [20–22, 24] measured the WOMAC function score to evaluate the short-term effect of laser acupuncture, and data from three [20, 21, 24] of the four studies evaluated the long-term effect. The pooled effect showed no significant difference between laser acupuncture and placebo at either time point (SMD = −0.28 (95% CI = −0.79, 0.23), *I*^2^ = 73%, *p*=0.28; SMD = −0.25 (95% CI = −0.78, 0.27), *I*^2^ = 70%, *p*=0.34) (Supplementary [Supplementary-material supplementary-material-1]). Besides, data on short-term and long-term WOMAC stiffness outcome were available in three [20–22] and two studies [20, 21], respectively. The combined effect also failed to identify any significant difference between two interventions (SMD = −0.19 (95% CI = −0.76, 0.37), *I*^2^ = 60%, *p*=0.50; SMD = −0.26 (95% CI = −0.68, 0.17), *I*^2^ = 4%, *p*=0.23) (Supplementary [Supplementary-material supplementary-material-1]). Furthermore, the outcome of quality of life was measured in three studies [21, 24, 25] by different assessment methods, and one study [25] reported positive result in the short-term effect of laser acupuncture. However, the overall effect demonstrated that there was no significant difference between therapies, no matter at which time point (SMD = −0.53 (95% CI = −1.40, 0.35), *I*^2^ = 90%, *p*=0.24; SMD = −0.11 (95% CI = −0.18, 0.39), *I*^2^ = 0%, *p*=0.46) (Supplementary [Supplementary-material supplementary-material-1]).

## 4. Discussion

The results of this systematic review and meta-analysis assessed the clinical effectiveness of laser acupuncture in the treatment of knee OA, mainly focusing on pain and functional outcomes. This study indicated that laser acupuncture only has the short-term effect for pain relief in terms of the VAS pain score, and this effect does not maintain for a long period; this finding is inconsistent with the conclusion from a previous meta-analysis [11], which reported that the effectiveness of laser acupuncture on the management of musculoskeletal pain tended to be more significant during long-term follow-up periods rather than at the end of intervention. Therefore, what needs to be emphasized is that the credibility of results from included studies could be related to a variety of factors. Subgroup analysis performed in this study showed that studies with appropriate technical features (both laser parameters applied and acupuncture points stimulated) or higher methodological quality tended to get a more significant result, with a favorable effect of laser acupuncture on both short-term and long-term pain outcomes, although the effect faded away over time. But up to now, there are less laser acupuncture research studies on the treatment of knee OA meeting the above two criteria, which makes the results from subgroup analysis less convincible. Except for the methodological quality and technical features, an adjunctive intervention might also influence the results. Among the included studies, Al Rashoud et al. [23] used exercise and advice as an additional treatment in both experimental and control groups, which might potentially magnify the role of laser acupuncture.

This updated review of laser acupuncture has its strengths and shortcomings. Strengths include several aspects as follows. Firstly, as we can see, all included seven studies were high in quality based on the PEDro score, and near half of them used the appropriate technical parameters according to the guideline recommendations or expert consensus. Secondly, unlike previous systematic review and meta-analysis of laser acupuncture [10, 11], this review focused on knee OA specially, making the results for both pain and functional outcome measures more consistent and precise. These all intensify the quality of evidence. Also, some shortcomings need further discussion. First and foremost is the potential bias resulting from high heterogeneity between the pooled studies, so the random-effects model was chosen and different subgroup analyses were performed. Second, the acupuncture points stimulated in the experimental groups were not diverse among the included studies although they all were selected by the experienced acupuncturists and were deemed appropriate. According to the Traditional Chinese Medicine (TCM) theory, each acupuncture point is distinguished by different energy flows and rhythms, and different points have specific and differentiated effects on knee OA patients [20]. However, there are difficulties in prescribing acupuncture points on a formulaic basis as part of a clinical trial to standardize treatment for all subjects [26]. Last, the baseline characteristics of recruited KOA patients are considered a typical host factor that might affect the intervention outcomes [5, 27]. Although the studies mainly enrolled patients with Kellgren–Lawrence grade II and III KOA, the detailed baseline levels of pain or function score were not available in the included studies (only mean ± SD known), causing that the subgroup analysis based on the severity of KOA could not be performed to eliminate this potential risks for the accuracy of the results.

## 5. Conclusions

On the basis of the results of the current review, laser acupuncture can effectively reduce the patient's subjective perception of knee pain at short term, but the effect likely fades away during the subsequent follow-up period, while it is unlikely to stop the progression of knee OA (according to the functional outcomes). Considering the discrepant results from subgroup analysis, future trials of laser acupuncture for knee OA should be designed with the more standardized laser parameters and the more consistent acupuncture point scheme. Furthermore, other more precise outcome measurement tools, such as inflammatory biomarkers or image study, should probably be introduced into future studies to make the evidence more convincing.

## Figures and Tables

**Table 1:** Summary of study characteristics.

Study	Participant characteristics	*n*	Interventions	Follow-up	Outcome measures	Results
Yurtkuran et al. [21]	1. KOA patients with Kellgren–Lawrence grades II and III 2. An average pain intensity of ≥40 on a 100 mm VAS over the last month before baseline assessment	52	Active laser acupuncture (27) vs. placebo (25)	2^nd^ week PI^a,b^; 12^th^ week	1. Pain—pVAS; WOMAC pain score 2. Function—50 foot w; WOMAC function score 3. Stiffness—WOMAC stiffness score 4. Others: KC; MTS; NHP	Laser acupuncture was found to be effective only in reducing periarticular swelling when compared with placebo
Shen et al. [22]	1. KOA patients with Kellgren–Lawrence grade ≥2 2. Moderate or greater clinically significant knee pain on most days during the previous month	35	Active laser acupuncture (19) vs. placebo (16)	2^nd^ week PI^b^	1. Pain—WOMAC pain score 2. Function—WOMAC function score 3. Stiffness—WOMAC stiffness score 4. Others: adverse effects	Laser acupuncture was found to be effective in improving WOMAC index scores for pain, stiffness, and function when compared with placebo
Al Rashoud et al. [23]	1. Patients with KOA according to the American College of Rheumatology criteria 2. An average pain intensity of ≥3 on a 10 cm VAS	49	Active laser acupuncture (26) vs. placebo (23)	After fifth session; after last session; 6 week PI^a^; 6 month PI	1. Pain—VAS 2. Function—SKFS score	Short-term application of LLLT to specific acupuncture points in association with exercise and advice is effective in reducing pain and improving quality of life in patients with KOA
Hinman et al. [24]	1. People who have knee pain of longer than 3 months' duration and morning stiffness lasting less than 30 minutes 2. An average pain severity of 4 or more out of 10 on an NRS	141	Active laser acupuncture (71) vs. placebo (70)	12^th^ weeks (8 week PI)^a,b^; 1 year	1. Pain—NRS; WOMAC pain score 2. Function—WOMAC function score 3. Others: AQoL-6D; SF-12	Laser acupuncture resulted in modest improvements in pain compared with control at 12 weeks that were not maintained at 1 year. But there were no significant differences in outcomes between active and sham laser acupuncture at 12 weeks or 1 year
Helianthi et al. [25]	1. KOA patients with Kellgren–Lawrence grades II and III 2. An average pain intensity of ≥40 on a 100 mm VAS	59	Active laser acupuncture (30) vs. placebo (29)	After 4 sessions; after 9 sessions^a^; 2 week PI	1. Pain—VAS 2. Others: Lequesne index	Laser acupuncture had a more effective effect on reducing VAS and Lequesne index in the elderly patients with KOA compared to placebo treatment
Suen et al. [19]	1. Patients with KOA according to the clinical criteria of the American College of Rheumatology guidelines 2. People who experienced knee pain	19	Laser acupuncture (10) vs. placebo (9)	6^th^ week PI^a^	1. Pain—NRS 2. Function—TUGT; the active and passive ROM of the knee	Nearly all outcome measures showed significant differences before and after intervention in subjects who received laser acupuncture treatment, but there are no significant differences between laser acupuncture and placebo groups
Rees et al. [20]	1. KOA patients with Kellgren–Lawrence grades II and III 2. People who suffer OA less than 10 years	40	Active laser acupuncture (20) vs. placebo (20)	4^th^ week PI^a^; 8^th^ week^b^; 12^th^ week	1. Pain—VAS; WOMAC pain score; SF-MPQ 2. Function—WOMAC function score 3. Stiffness—WOMAC stiffness score 4. Others: WAI-C; MHLC-C	Laser acupuncture can safely reduce OAK pain and stiffness and improve physical function

^a^Time point of short-term VAS pain outcome measures in the included studies. ^b^Time point of short-term WOMAC pain outcome measures. *n*, sample size; vs., versus; pVAS, pain on motion with visual analogue scale; 50 foot w, 50 foot walking distance; KC, knee circumference; MTS, medial tenderness score of the knee; NHP, Nottingham Health Profile total score; SKFS, Saudi Knee Function Scale; PI, postintervention; NRS, numeric rating scale; AQoL-6D, Assessment of Quality of Life instrument version 2; SF-12, 12-item Short-Form Health Survey; TUGT, time up and go test; ROM, range of movement; SF-MPQ, Short-Form McGill Pain Questionnaire; WAI-C, Working Alliance Inventory short form; MHLC-C, Multidimensional Health Locus of Control short form C.

**Table 2:** Technical features of laser acupuncture treatments in the included studies.

Study	Laser type	Irradiated acupuncture points	Treatment time/no. of total sessions/no. of sessions per week	Output power (mW)	Energy density (J/cm^2^)	Energy per acupoints (J/point per session)	Comment
Yurtkuran et al. [21]	GaAs 904 nm	SP9—Yinlingquan	120 s/10/5	4	1.2	0.48	Acupuncture points: appropriate, but limitedLaser parameters: inappropriate, for the dosage is too low
Shen et al. [22]	A 650 nm semiconductor laser combined with a 10.6 *μ*m CO_2_ laser	ST35—Dubi	20 min/12/3	36 and 200, respectively	NA	NA	Acupuncture points: appropriate, but limitedLaser parameters: inappropriate, for the 10.6 *μ*m CO_2_ laser has the thermal effect
Al Rashoud et al. [23]	GaAs 830 nm	SP9—Yinlingquan, SP10—Xuehai, ST36—Zusanli, ST-35—Dubi, medial Xiyan	40 s/9/3	30	4	1.2	Acupuncture points: appropriateLaser parameters: inappropriate, for the dosage is too low
Hinman et al. [24]	NA	Selected by acupuncturists depending on clinical examination	20 min/8/2	10	NA	0.2	Acupuncture points: appropriateLaser parameters: inappropriate, for the dosage is too low
Helianthi et al. [25]	GaAlAs 785 nm	ST35—Dubi, ST36—Zusanli, SP9—Yinlingquan, GB34—Yanglingquan, EX-LE-4—Neixiyan	80 s/10/2	50	NA	4	Acupuncture points: appropriateLaser parameters: appropriate
Suen et al. [19]	650 nm	TF4—“shenmen” AH4—“knee” CO13—“spleen” CO12—“liver” CO10—“kidney” AT4—“subcortex”	60 s/18/3	2.5	0.54	NA	Acupuncture points: appropriateLaser parameters: appropriate
Rees et al. [20]	GaAs 810 nm	13 points based on presenting syndrome	26 min/12/3	100	74.4	18	Acupuncture points: appropriateLaser parameters: appropriate

NA, not available.

**Table 3:** Summary of the meta-analysis results for secondary outcomes.

Parameters	Measure points	SMD (95% CI)^*∗*^	*I* ^2^ (%)	*p*
WOMAC function score outcome	Short-term	−0.28 (−0.79, 0.23)	73	0.28
	Long-term	−0.25 (−0.78, 0.27)	70	0.34
WOMAC stiffness score outcome	Short-term	−0.19 (−0.76, 0.37)	60	0.50
	Long-term	−0.25 (−0.67, 0.16)	4	0.23
Quality of life outcome	Short-term	−0.53 (−1.40, 0.35)	90	0.24
	Long-term	0.11 (−0.18, 0.39)	0	0.46

^*∗*^Random-effects model was used when *I*^2^ ≥ 50%; otherwise, fixed-effects model was used.

## Supplementary Materials

Supplementary Materials Supplementary Table 1: search strategy—Ovid format. Supplementary Table 2: summary of study quality. Supplementary Figure 1: forest plots of the LAT effects on short-term pain relief. Supplementary Figure 2: forest plots of the LAT effects on functional outcome. Supplementary Figure 3: forest plots of the LAT effects on stiffness outcome. Supplementary Figure 4: forest plots of the LAT effects on quality of life.
