# Supplementary material for: Laser Acupuncture for Patients with Knee Osteoarthritis: A Systematic Review and Meta-Analysis of Randomized Placebo-Controlled Trials
Source: Evid Based Complement Alternat Med. 2019 Nov 3;2019:6703828. doi: 10.1155/2019/6703828 (PMC6874873; doi:10.1155/2019/6703828)
Supplement: Supplementary Materials — Supplementary Table 1: search strategy—Ovid format. Supplementary Table 2: summary of study quality. Supplementary Figure 1: forest plots of the LAT effects on short-term pain relief. Supplementary Figure 2: forest plots of the LAT effects on functional outcome. Supplementary Figure 3: forest plots of the LAT effects on stiffness outcome. Supplementary Figure 4: forest plots of the LAT effects on quality of life. [file 6703828.f1.docx]

# Supplementary

## Supplementary Table 1. Search strategy: Ovid format

| Phase 1 | Phase 2 | Phase 3 |
| --- | --- | --- |
| 1. acupunc*.ti,ab,kw. 2. exp Acupuncture/ 3. acupoint*.ti,ab,kw. 4. exp Acupuncture Point~~s~~/ 5. exp Acupuncture Therapy/ 6. trigger.ti,ab,kw. 7. exp Trigger Point~~s~~/ 8. or/1-7 9. therap*.ti,ab,kw. 10. treatment.ti,ab,kw. 11. or/9,10 12. and/8,11 13. exp Low Level Laser Therapy/ 14. laser*.ti,ab,kw. 15. LLLT.ti,ab,kw. 16. or/13-15 17. 12 and 16 | 1. OA.ti,ab,kw. 2. osteoarthr*.ti,ab,kw. 3. exp Osteoarthritis/ 4. arthrosis.ti,ab,kw. 5. Chronic Pain/ 6. or/18-22 7. Knee/ 8. exp Knee Joint/ 9. knee$.ti,ab,kw. 10. or/24-26 11. 23 and 27 12. exp Osteoarthritis, Knee/ 13. 28 or 29 14. 17 and 30 | 1. exp Clinical Trial/ 2. clinical trial.ab. 3. exp Research Design/ 4. research design.ab. 5. Random Allocation/ 6. random*.ab. 7. Double-Blind Method/ 8. Single-Blind Method/ 9. blind*.ab. 10. placebo*.ab. 11. Placebos/ 12. or/32-42 13. Human/ 14. 43 and 44 15. 31 and 45 |

# Supplementary Table 2. Summary of study quality

| **Study** | | | PEDro criteria* | | | | | | | | | | | | | **Score** |
| --- | --- | --- | --- | --- | --- | --- | --- | --- | --- | --- | --- | --- | --- | --- | --- | --- |
|  |  |  | (1) | | 2 | 3 | 4 | 5 | 6 | 7 | 8 | | 9 | 10 | 11 |  |
| **Yurtkuran et al., 2007** | | | Y | | Y | Y | Y | Y |  | Y | Y | |  | Y | Y | **8** |
| **Shen et al., 2009**  **Al Rashoud et al., 2014**  **Hinman et al., 2014**  **Helianthi et al., 2016**  **Suen et al., 2016**  **Rees, 2017** | | | Y  Y  Y  Y  Y  Y | | Y  Y  Y  Y  Y  Y | Y  Y  Y  Y  Y  Y | Y  Y  Y  Y  Y  Y | Y  Y  Y  Y  Y  Y | Y | Y  Y  Y  Y  Y  Y | Y  Y  Y | | Y  Y  Y | Y  Y  Y  Y  Y  Y | Y  Y  Y  Y  Y  Y | **7**  **7**  **8**  **8**  **9**  **10** |
| *PEDro criteria: | | 4 | | Baseline comparability | | | | | | 8 | | Adequate follow-up | | | | |
| (1) | Eligibility criteria | 5 | | Blind subjects | | | | | | 9 | | Intention-to-treat analysis | | | | |
| 2 | Random allocation | 6 | | Blind therapists | | | | | | 10 | | Between-group comparisons | | | | |
| 3 | Concealed allocation | 7 | | Blind assessors | | | | | | 11 | | Point estimates and variability | | | | |
| Y – Criteria met; (1) – Eligibility criteria item does not contribute to total score | | | | | | | | | | | | | | | | |

# Supplementary Figure 1.


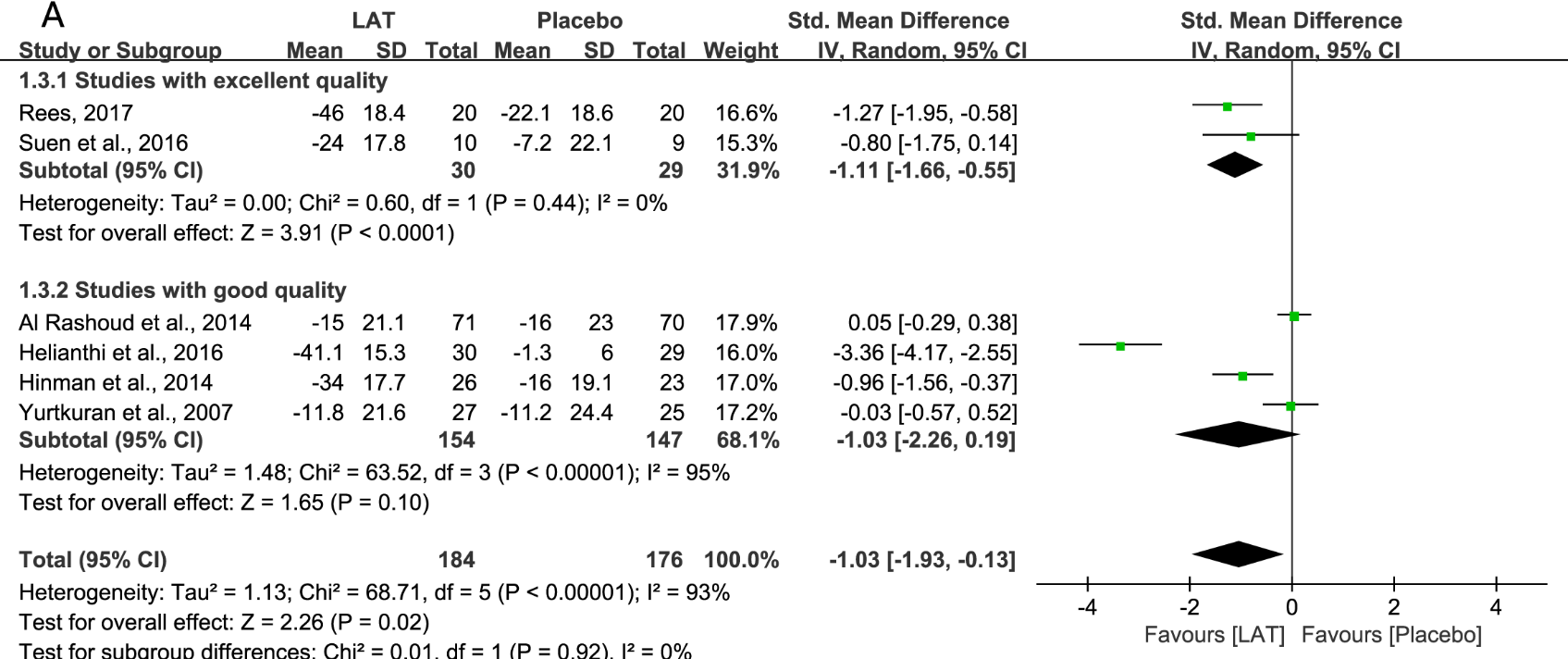

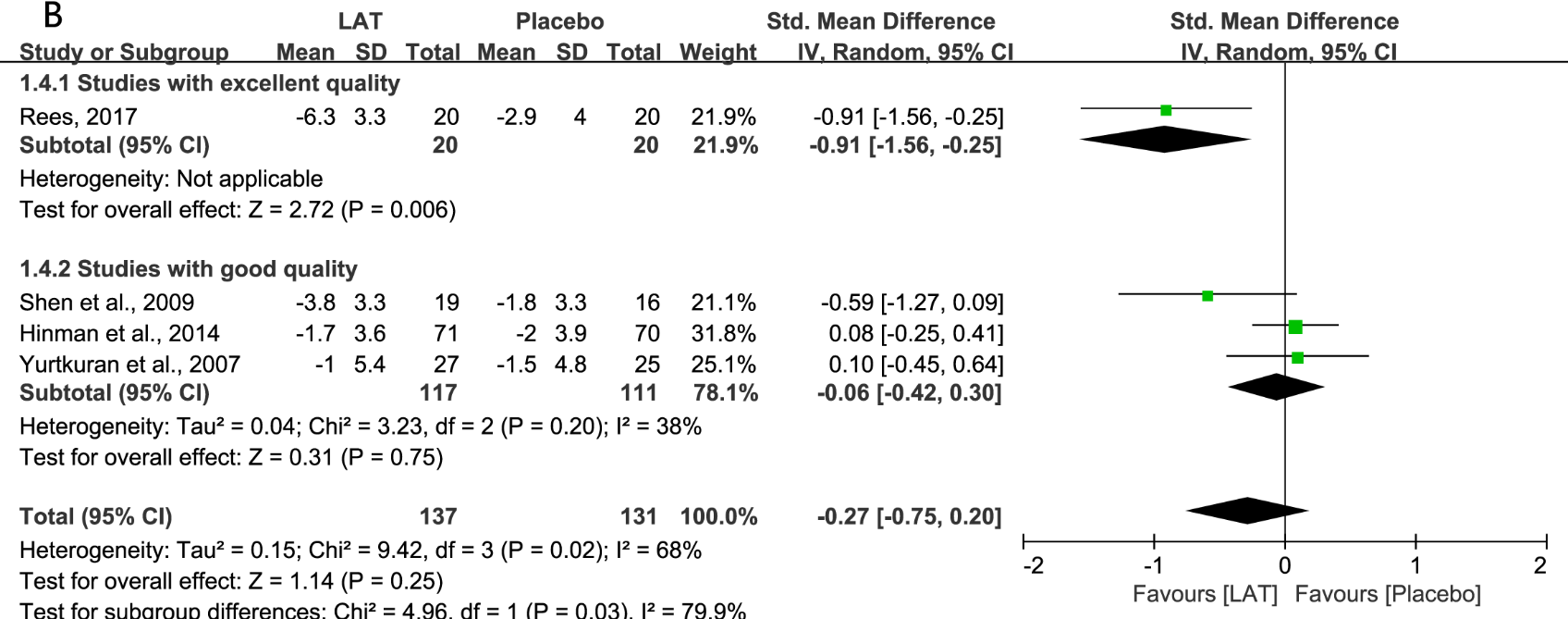


**Supplementary Figure 1.** Forest plots of the LAT effects on short-term pain relief. (**A**) Pain relief regarding the VAS pain score (subgroup analysis based on methodologic quality). (**B**) Pain relief regarding the WOMAC pain score (subgroup analysis based on methodologic quality). **Abbreviations:** LAT, laser acupuncture treatment; CI, confidence interval; SD, standard deviation.

# Supplementary Figure 2.


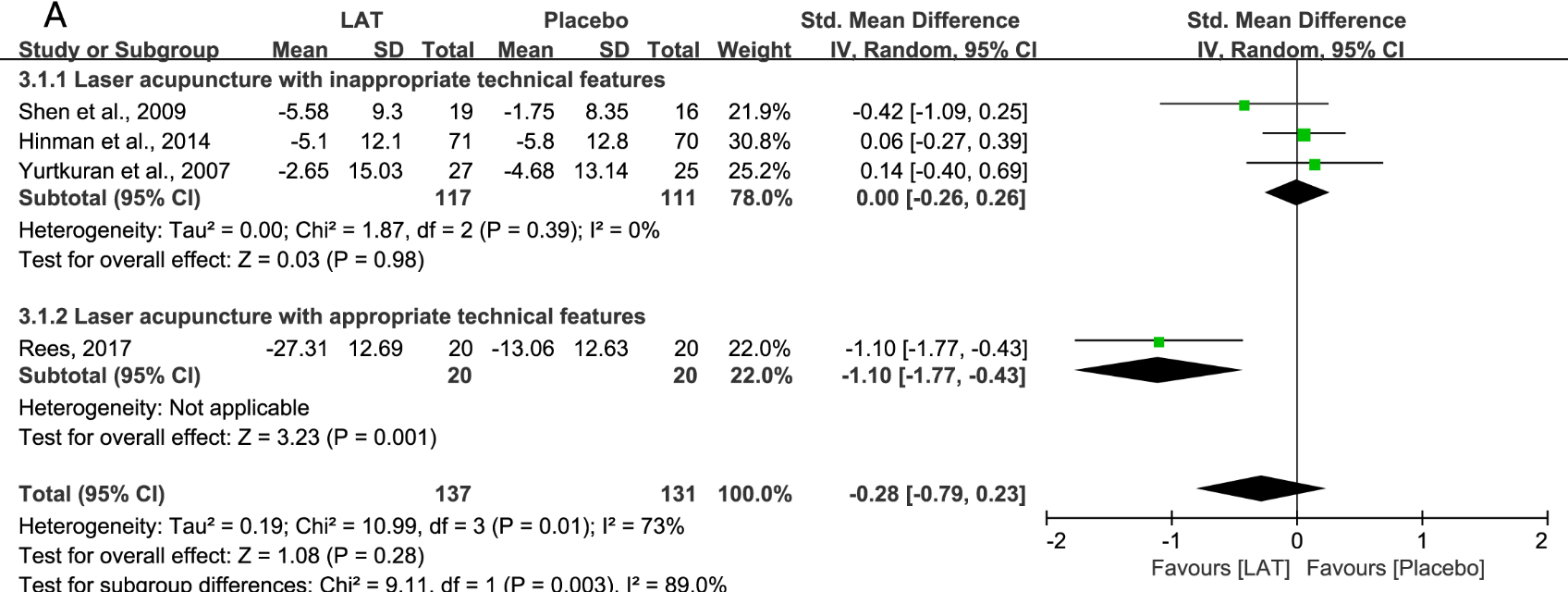


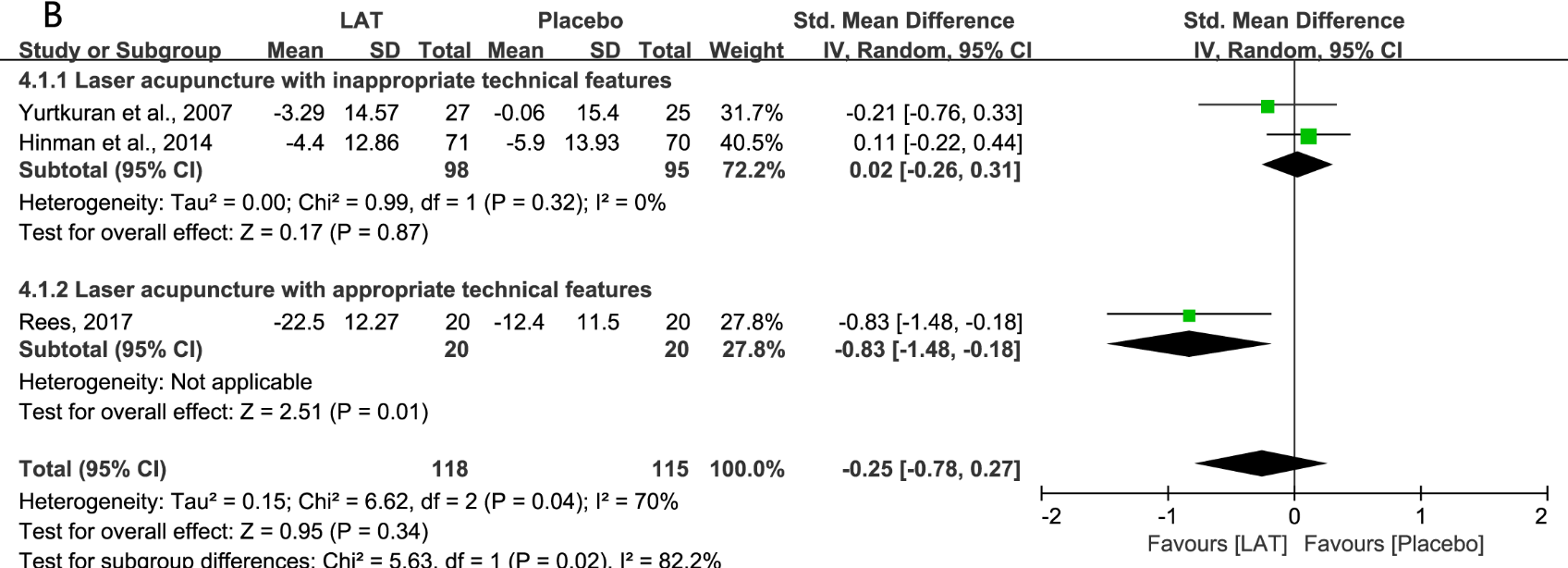


**Supplementary Figure 2.** Forest plots of the LAT effects on function outcome. (**A**) Short-term WOMAC function score (subgroup analysis based on whether studies with appropriate technical features). (**B**) Long-term WOMAC function score (subgroup analysis based on whether studies with appropriate technical features). **Abbreviations:** LAT, laser acupuncture treatment; CI, confidence interval; SD, standard deviation.

# Supplementary Figure 3.


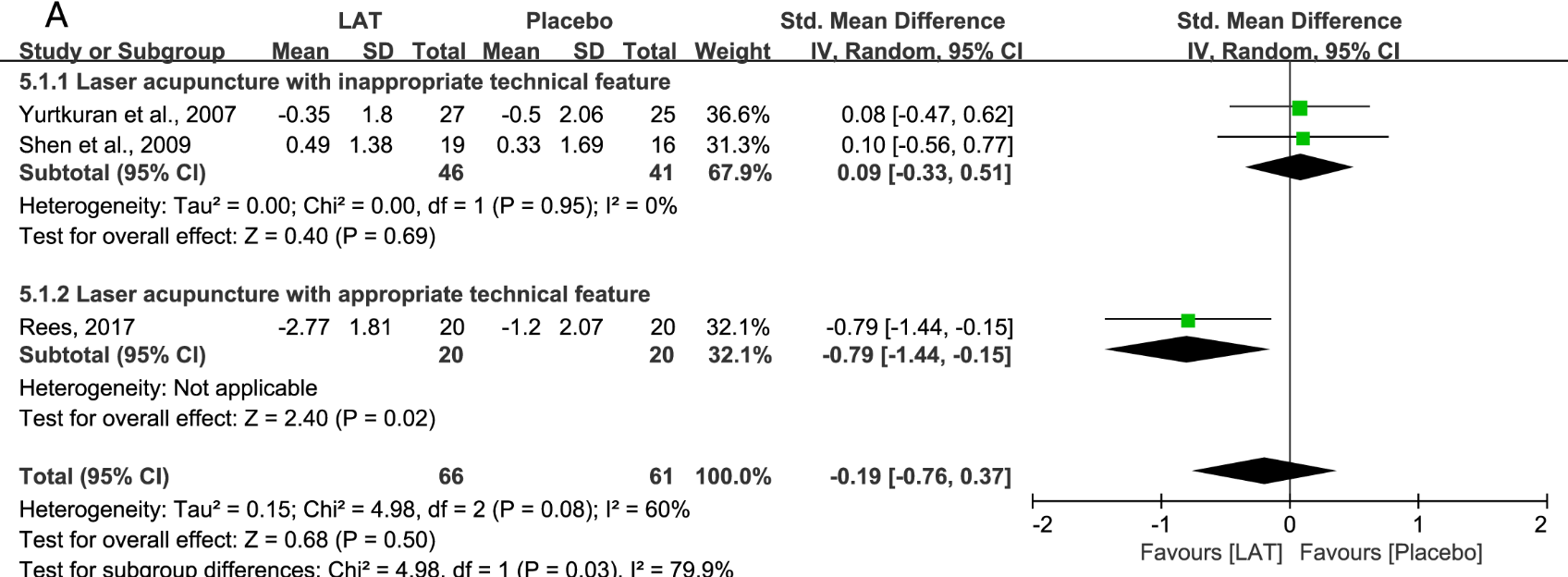

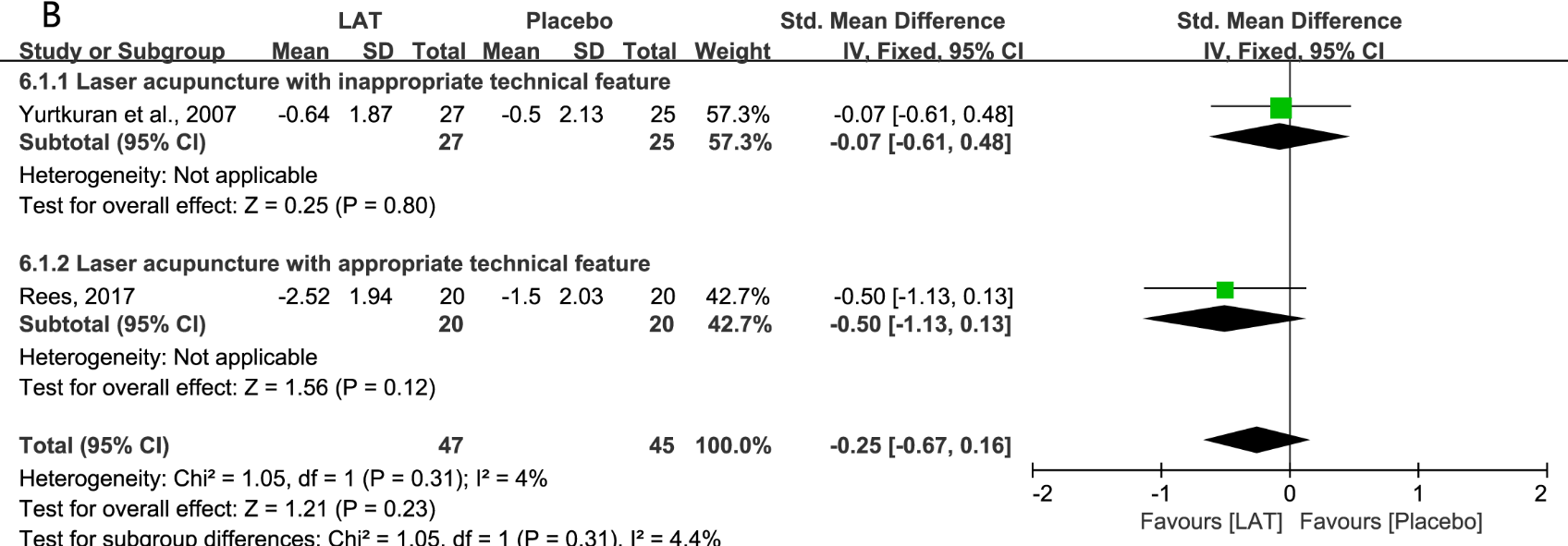


**Supplementary Figure 3.** Forest plots of the LAT effects on stiffness outcome. (**A**) Short-term WOMAC stiffness score (subgroup analysis based on whether studies with appropriate technical features). (**B**) Long-term WOMAC function score (subgroup analysis based on whether studies with appropriate technical features). **Abbreviations:** LAT, laser acupuncture treatment; CI, confidence interval; SD, standard deviation.

# Supplementary Figure 4.


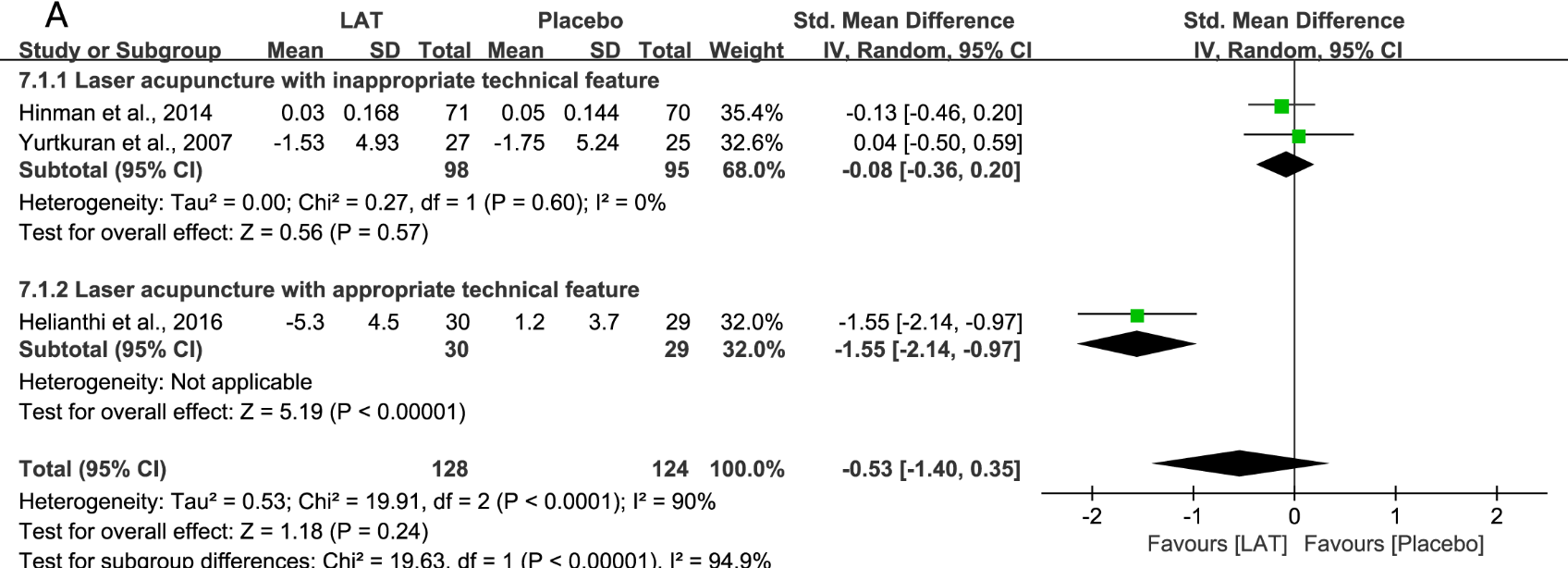

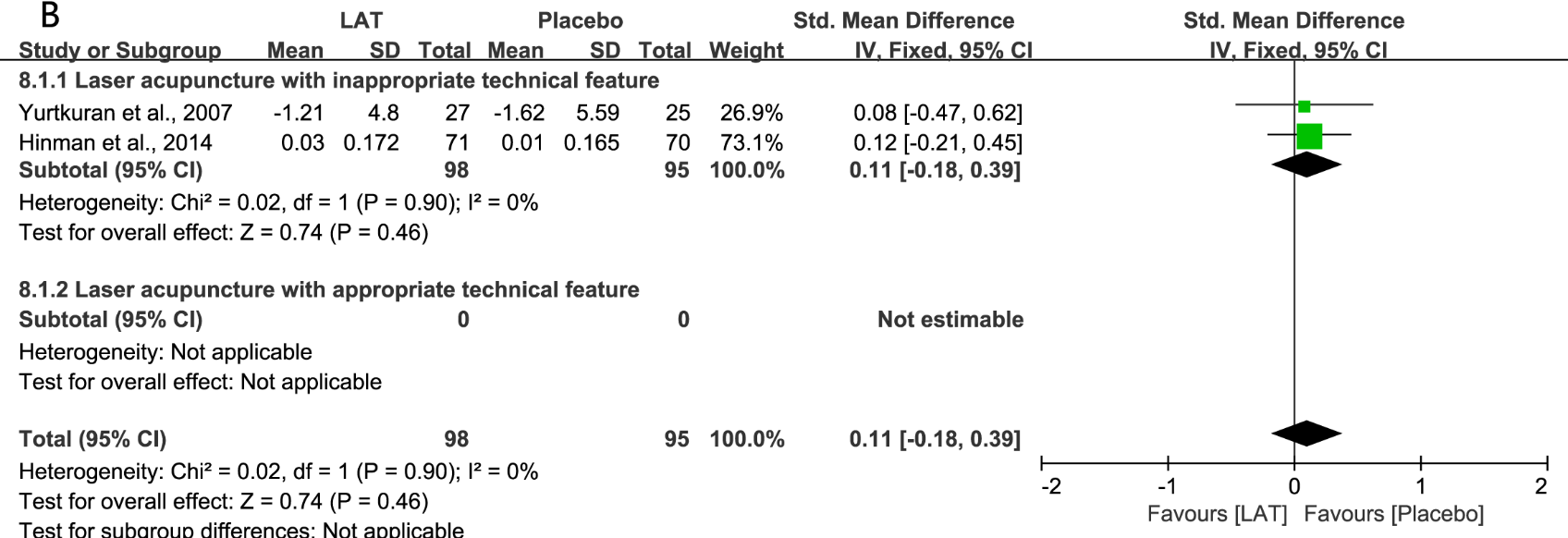


**Supplementary Figure 4.** Forest plots of the LAT effects on quality of life. (**A**) Short-term quality of life outcome (subgroup analysis based on whether studies with appropriate technical features). (**B**) Long-term quality of life outcome (subgroup analysis based on whether studies with appropriate technical features). **Abbreviations:** LAT, laser acupuncture treatment; CI, confidence interval; SD, standard deviation.
